# Thermochemical investigations in the system Cd–Gd

**DOI:** 10.1016/j.jallcom.2014.05.062

**Published:** 2014-10-15

**Authors:** Thomas L. Reichmann, Rajesh Ganesan, Herbert Ipser

**Affiliations:** a Department of Inorganic Chemistry (Materials Chemistry), University of Vienna, 1090 Vienna, Austria; b Indira Gandhi Centre for Atomic Research, Kalpakkam, Tamil Nadu, India

**Keywords:** Cd–Gd alloys, Isopiestic measurements, Vapour pressure method, Thermodynamic properties

## Abstract

Vapour pressure measurements were performed in terms of a non-isothermal isopiestic method to determine vapour pressures of Cd in the system Cd–Gd between 693 and 1045 K. From these results thermodynamic activities of Cd were derived as a function of temperature for the composition range 52–86 at.% Cd. By employing an adapted Gibbs–Helmholtz equation, partial molar enthalpies of mixing of Cd were obtained for the corresponding composition range, which were used to convert the activity values of Cd to a common average sample temperature of 773 K. The relatively large variation of the activity across the homogeneity ranges of the phases Cd_2_Gd and Cd_45_Gd_11_ indicates that they probably belong to the most stable intermetallic compounds in this system.

An activity value of Gd for the two phase field Cd_6_Gd+L was available from literature and served as an integration constant for a Gibbs–Duhem integration. Integral Gibbs energies are presented between 51 and 100 at.% Cd at 773 K, referred to Cd(l) and α-Gd(s) as standard states. Gibbs energies of formation for the exact stoichiometric compositions of the phases Cd_58_Gd_13_, Cd_45_Gd_11_, Cd_3_Gd and Cd_2_Gd were obtained at 773 K as about −19.9, −21.1, −24.8, and −30.0 kJ g atom−^1^, respectively.

## Highlights

• Vapour pressures of Cd were determined in Cd-Gd alloys.
• Thermodynamic activities of Cd are given between 52 and 86 at.% Cd.
• Enthalpies of mixing of Cd were determined as a function of composition.
• Gibbs energies of formation are given at 773 K.

## 1 Introduction

In the last decades, the question of how to satisfy the ever increasing demand of energy has become most pressing for countries with high economic growth. The utilization of nuclear energy is sometimes inevitable for nations belonging to the group of developing and emerging countries. For an efficient use of nuclear energy, these countries have to establish strategies comprising an optimized reprocessing routine of spent nuclear fuels as well as an adequate waste management on the back-end of their nuclear fuel cycle. Indeed, low-level and intermediate-level radioactive waste is currently stored in interim storage facilities or deposited in geological repositories. Solutions for high-level waste are currently still in a planning stage and thus this type of waste is actually stored on-site.

Focusing onto reprocessing of nuclear waste, electro-transport and reductive extraction is applied to separate actinides and lanthanides from high-level radioactive waste (cf. Refs. [1–5]). Moriyama et al. [6] determined that the separation factors, which are an indicator for extractability, are quite different between actinides and lanthanides and are predominantly dependent on the employed liquid metal which is preferentially Cd [7]. Therefore, a detailed knowledge of the respective Cd-RE phase diagrams as well as of the thermodynamic stabilities of the corresponding intermetallic compounds is of great importance. This was the reason for initiating a series of thermodynamic and phase diagram studies of different Cd-RE systems (cf. Refs. [8–10]).

Concerning the Cd–Gd phase diagram, a rather complete description was given by Bruzzone et al. [11], who applied conventional methods, i.e. differential thermal analysis and chemical-, metallographic- and X-ray analyses. Six intermetallic compounds in total were determined, i.e. CdGd, Cd_2_Gd, Cd_3_Gd, Cd_45_Gd_11_, Cd_58_Gd_13_ and Cd_6_Gd, where for Cd_2_Gd a polymorphic transformation into a high temperature modification was indicated at 995 °C. All intermetallic compounds were shown to be line compounds, i.e. no significant solid solubility of either Cd or Gd is indicated.

Besides the work of Bruzzone et al. [11], only limited information concerning phase equilibria was available from literature. Johnson [12] reported liquidus data in the temperature range 324–500 °C, determined by chemical analysis of filtered samples of corresponding equilibrium phases. Tang and Gschneidner [13] prepared single-phase samples located within the homogeneity range of the Cd stabilized high temperature modification of Gd (β-Gd). Using differential thermal analyses (DTA) these authors could determine that the eutectoid decomposition of β-Gd takes place at 738 °C whereas Bruzzone et al [11] reported a value given as 725 °C.

Although a rather complete description of the phase diagram was available, less information was found in literature regarding thermodynamic data. In an early work Roshchina and Bayanov [14] employed an electrochemical technique to determine activities of Gd in liquid Cd between 390 and 530 °C. By means of a Gibbs–Duhem integration, the authors were able to calculate the standard Gibbs energy of formation of Cd_6_Gd, given as −20.8 ± 0.6 kJ mol(at)^−1^, referred to the elements in their standard states.

In another early study Kurata et al. [15] presented an activity coefficient of Gd in liquid Cd at 500 K, given as −6.20. It was stated that this value was derived from an evaluation of electrochemical studies of Sakamura et al. [16]. These latter authors listed activity coefficients of rare earth elements in liquid Cd at 450 °C. Their corresponding activity coefficient of Gd in liquid Cd at 450 °C was −6.60.

Based on the results of Refs. [11,12,16], Kurata and Sakamura [17] made a CALPHAD-type optimization of the complete Cd–Gd system. All intermetallic compounds were modelled as stoichiometric line compounds, and temperature dependent Gibbs energies were listed which could be used for comparison in the present study.

## 2 Experimental

**Fig. 1:**
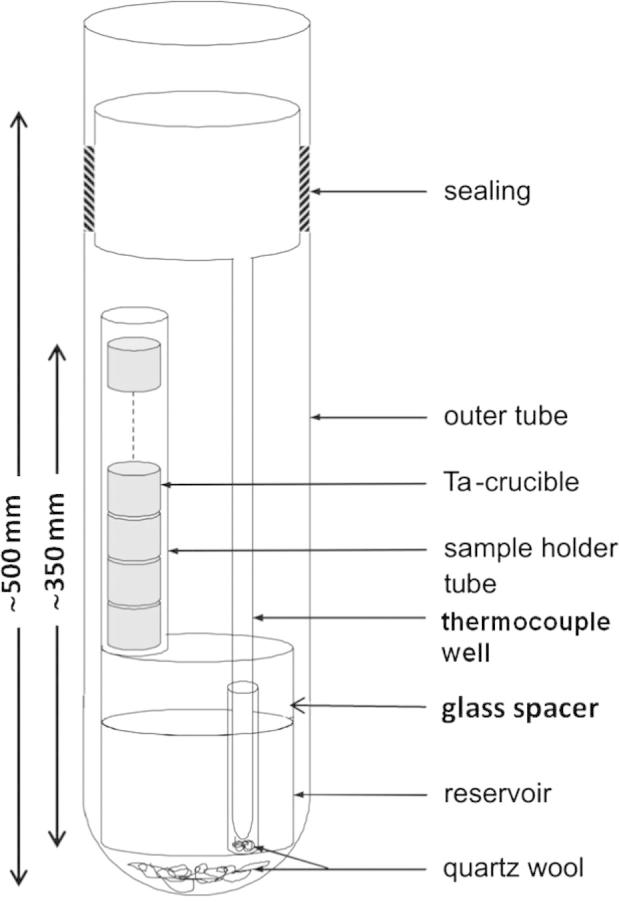
Isopiestic quartz glass apparatus used in the present study.

A non-isothermal isopiestic method was applied to determine Cd vapour pressures in Cd–Gd alloys. This method was previously described by Ipser et al. [18]. The corresponding experimental arrangement is shown schematically in Fig. 1. The entire setup is made of fused silica glass and consists of four parts. A silica glass crucible, where the volatile metal is held (the reservoir), is placed at the bottom of the outer tube. On top of this crucible a glass spacer is located, connected to a sample holder tube, in which tantalum crucibles are stacked one on top of the other. The defined distance from the bottom of the reservoir to the uppermost crucible is around 350 mm. Another inner tube with its upper end widened is used as a thermocouple well. This widened part is sealed with the outer tube under dynamic vacuum.

Before use, the entire apparatus was cleaned with an acid mixture (HF/HNO_3_/H_2_O), rinsed with distilled water and dried. Afterwards the completely assembled setup, including the empty Ta crucibles (approximately 20), was degassed under dynamic vacuum (10^−3^ mbar) at 900 °C for 5 h. Depending on the experimental temperatures, the reservoir was filled with 25–35 g Cd (99.9999% Alfa AESAR, Karlsruhe, Germany). All further handling was carried out in a glove box, filled with Ar (oxygen level: <1 ppm, water level: <1 ppm), to protect the Gd samples from oxidation.

At the beginning, several experiments were carried out using tiny pieces of pure Gd (99.9 %, Smart Elements, Vienna, Austria) as sample material, but it became obvious that during equilibration of Gd with Cd vapour the diffusion is rather low, i.e. no intermetallic compounds were formed within the samples. Although samples were equilibrated at different temperatures, it was not possible to reach equilibrium states within any reasonable time. In a second series of experiments Gd powder (99.9% Alfa AESAR, Karlsruhe, Germany) was employed as sample material. Although first results appeared to be encouraging it was observed that powder X-ray diffraction (XRD) patterns, obtained from the pure Gd powder, showed predominantly the presence of Gd_2_O_3_ together with traces of Gd(OH)_3_ whereas the expected reflections from Gd itself were not obtained. It must be assumed that the Gd particles in the powder are coated with a rather dense oxide/hydroxide layer. Obviously, this layer and relevant absorption effects due to the high atomic number of Gd are the reason for the almost undetectable intensity of Gd reflections.

Therefore it was finally decided to use a master alloy as sample material for the vapour pressure measurements, which was produced from Cd and Gd bulk material (99.9999% Alfa AESAR, Karlsruhe, Germany; 99.9%, Smart Elements, Vienna, Austria). Several attempts were carried out to synthesize single-phase alloys from different Cd–Gd intermetallic compounds. The best results were achieved for the compound Cd_45_Gd_11_. Stoichiometric amounts of Cd and Gd were weighed into a Mo crucible to a total amount of 12 g which was sufficient for two isopiestic runs. The crucible was covered with a Mo lid and enclosed into a silica glass tube under dynamic vacuum. The arrangement was slowly heated to 750 °C, a temperature below the boiling point of Cd, and held for two days. Afterwards, the alloy was cooled within four days to 525 °C, held for three more days and then cooled to ambient temperature. The rather low cooling rate was selected to guarantee a continuous reduction of the Cd vapour pressure within the crucible and to prevent condensation of Cd on the crucible wall. The master compound was powdered and characterized by XRD to be phase pure. About 200–350 mg of the Cd_45_Gd_11_ master alloy was weighed into each Ta crucible (10 mm o.d., 12 mm height) with an accuracy of ±0.1 mg.

After loading the arrangement (Fig. 1), it was securely closed with a glass stopper and shuttled out of the glove box. It was connected to a vacuum pump, evacuated and sealed under a dynamic vacuum of better than 10^−4^ mbar.

In the following, the isopiestic experiments were heated in different temperature gradients using two-zone furnaces. A total number of seven runs were performed at different reservoir (*T*_R_) and sample temperatures (*T*_S_). The respective temperature gradients were recorded by raising a Pt/Pt10%Rh thermocouple inside the thermocouple well. Each experiment lasted for about four to six weeks, depending on the reservoir temperature. After equilibration, the isopiestic apparatus was quenched in cold water and cut open by a diamond saw. The Cd–Gd alloys which had been formed within the Ta crucibles during equilibration with the Cd vapour were weighed back and their compositions calculated from the difference in weight, which was attributed to the uptake of Cd.

Representative samples were characterized by powder XRD with Cu Kα radiation using a Bruker D8 Advance Diffractometer with Bragg-Brentano geometry. To protect the powdered alloys from oxidation, a special XRD sample holder with an X-ray transparent lid was used. The corresponding XRD patterns were analyzed and refined by means of TOPAS 3 software (provided by Bruker), applying the fundamental parameter approach for peak profile modelling.

**Fig. 2:**
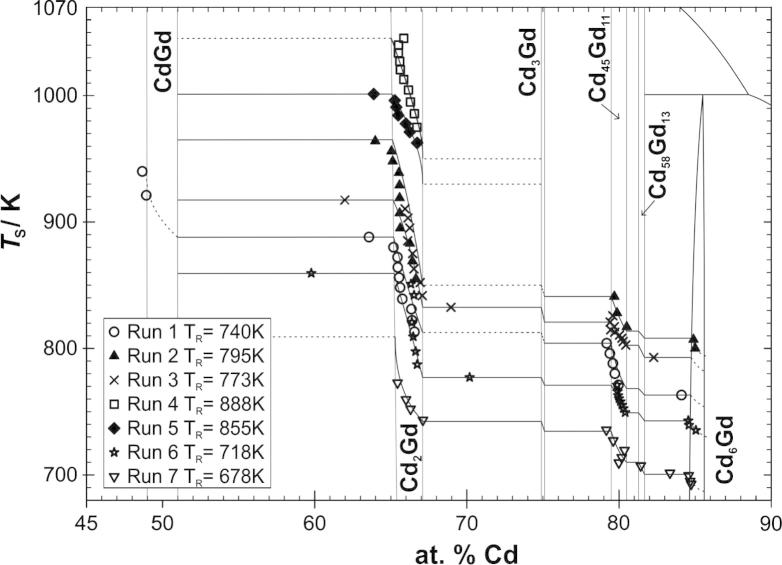
Sample temperature against sample composition superimposed on the partial Cd–Gd phase diagram [11]. Assumed trends of the equilibrium curves are drawn with dotted lines.

In an attempt to determine accurate sample compositions, selected alloys were dissolved in nitric acid and analysed spectroscopically. For the determination of the Cd content a PerkinElmer AAS with flame atomizer (AAnalyst 200) was used with an accuracy of about ±0.01 mg/L. The Gd content was measured with an ICP-MS (Agilent Technologies 7500ce) and an accuracy of ±0.01 μg/L. Unfortunately, the derived compositions showed a considerable scatter with a very slight tendency to be somewhat richer in Cd. Based on this scatter, it had to be concluded that the overall error for the results from AAS and ICP-MS was in the range of ±1 at.% Cd. This rather high error is probably due to the number of dilution steps of the aqueous solutions of the alloys which were necessary to achieve concentrations within the dynamic ranges of AAS and ICP-MS measurements. Considering this significant error, it was decided to use the alloy compositions as derived from weighing. Nevertheless, it is possible that the actual compositions during equilibration of the alloys are indeed slightly richer in Cd and that, due to the finite quenching rate, some of the Cd re-evaporates. This behaviour is outlined in detail in Ref. [8]. Considering all this, the resulting error in compositions was estimated not to exceed ±0.5 at.%. This was kept in mind when empirically fitting our isopiestic data in terms of the equilibrium curves which are shown in Fig. 2.

## 3 Results and discussion

### 3.1 Isopiestic experiment

On the basis of seven isopiestic runs which were carried out at different temperature conditions, thermodynamic activities were derived as a function of temperature and composition in the range between 51 and 86 at.% Cd. The reservoir temperatures which were set between 678 and 888 K correspond to Cd vapour pressures between 2 and 137 mbar, respectively. All samples were placed within a temperature interval of 693–1045 K. The vapour pressure of pure Cd at different temperatures was calculated according to the equation of Binnewies and Milke [19]:

(1) $$\log\left(\frac{p_{\text{Cd}}^{0}}{\text{bar}}\right)=8.7-5690\cdot \frac{\text{K}}{T}-1.07\cdot \log\frac{T}{\text{K}}$$

Since the melting point of Cd is rather low and it has indeed a much higher vapour pressure compared to Gd at the respective experimental temperatures, the total pressure in the system is determined only by the temperature of the Cd reservoir (*T*_R_). Equilibrium within the system is reached when the Cd vapour pressure over each sample, at its corresponding sample temperature *T*_S_, is equal to the vapour pressure of pure Cd at the reservoir temperature *T*_R_:

(2) $$p_{\text{Cd}}(T_{\text{S}})=p_{\text{Cd}}^{0}(T_{\text{R}})$$

Thus the thermodynamic activity of Cd for each sample can be obtained combining Eqs. (1) and (2), based on the conventional definition for the activity:

(3) $$a_{\text{Cd}}(T_{\text{S}})=\frac{p_{\text{Cd}}(T_{\text{S}})}{p_{\text{Cd}}^{0}(T_{\text{S}})}=\frac{p_{\text{Cd}}^{0}(T_{\text{R}})}{p_{\text{Cd}}^{0}(T_{\text{S}})}$$

All results, including sample temperatures and compositions, sample identifications, Cd activities and partial enthalpy values, are listed in Table 1. The compositions were calculated from the mass change during the experiment as described above. All samples were ascribed to one- or two-phase fields in the phase diagram which was confirmed by powder XRD.

The relative uncertainty in the compositions due to experimental errors should not exceed 0.5 at.% as outlined in Section 2 and the measured temperatures are assumed to be accurate within ±2 K. (A more detailed discussion on possible error sources in this type of isopiestic experiments can be found in Ref. [18].)

From the derived sample compositions homogeneity ranges were estimated for all phases at 773 K (see Table 2). Except for Cd_58_Gd_13_ and Cd_3_Gd, all other compounds show significant homogeneity ranges. As indicated above, phase equilibria of all samples were identified by powder XRD and agree excellently with the defined homogeneity ranges.

By plotting sample compositions against sample temperatures *T*_S_ for one particular run, a so-called equilibrium curve can be drawn connecting the individual data points in terms of an isobar. Fig. 2 shows these equilibrium curves for all runs, superimposed on the Cd–Gd phase diagram version from Ref. [11] but corrected with the estimated homogeneity ranges from the present study. As can be seen, most of the samples are concentrated within the homogeneity ranges of the two phases Cd_2_Gd and Cd_45_Gd_11_, suggesting that these phases are among the most stable ones in the Cd–Gd system. Besides, no samples were obtained in the phase Cd_3_Gd suggesting that Cd_3_Gd is only slightly more stable than a two-phase mixture of its neighbouring compounds. This phenomenon was actually observed already earlier in the systems Cd–Pr and Cd–Ce where similarly no single-phase samples of the corresponding phases Cd_3_Pr and Cd_3_Ce were formed [8,9]. Nevertheless, it should be mentioned that Cd_3_Gd crystallizes in a different crystal structure compared to Cd_3_Pr and Cd_3_Ce [20], a fact that makes the comparability regarding stability of these phases more surprising.

As can be seen in Fig. 2, some of the samples formed in the isopiestic runs were obtained in various two-phase fields after equilibration. This is probably caused by slight variations of the sample temperatures. Nevertheless, these samples allow the calculation of Cd activities for the respective two-phase fields at the corresponding sample temperatures *T*_S_ (cf. Section 3.2).

### 3.2 Partial enthalpy of mixing of Cd in two-phase fields

**Fig. 3:**
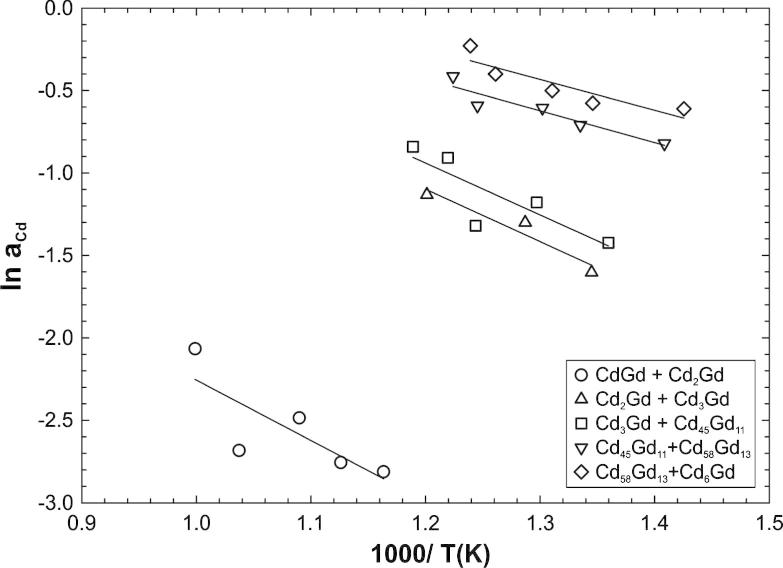
Natural logarithm of the Cd activity against reciprocal sample temperature for different two-phase fields.

From the Cd vapour pressures of samples located within two-phase fields, Cd activities were calculated at the respective sample temperatures *T*_S_ (see Table 1). Natural logarithms of these activity values were plotted against reciprocal sample temperatures for almost all two-phase fields, i.e.: CdGd + Cd_2_Gd, Cd_2_Gd + Cd_3_Gd, Cd_3_Gd + Cd_45_Gd_11_, Cd_45_Gd_11_ + Cd_58_Gd_13_ and Cd_58_Gd_13_ + Cd_6_Gd (see Fig. 3). According to an adapted Gibbs–Helmholtz equation (4), partial enthalpy values of mixing of Cd were directly calculated from the slopes:

(4) $$\frac{\partial \ln a_{\text{Cd}}}{\partial (1/T)}=\frac{\Delta {\overset{¯}{H}}_{\text{Cd}}}{R}$$

where the temperature is in K, *R* is the gas constant in J (mol K)^−1^, and the partial enthalpy of Cd is in J mol(Cd)^−1^. For this approach straight phase boundaries, i.e. no variation of solid solubilities with temperature, of the different compounds were assumed. Although it is obvious that the solubilities will be temperature dependent in the examined temperature ranges, this is still a useful approximation to obtain information on the corresponding partial enthalpy values in a certain temperature range within two-phase fields (cf. Table 1). By comparing the slopes in Fig. 3, an exothermic behaviour is observed in the corresponding composition range 50–86 at.% Cd. Obviously, the partial molar enthalpy of mixing of Cd becomes more negative with decreasing Cd content of the alloys. The relatively high difference of the Cd activities between two-phase fields adjacent to Cd_2_Gd and Cd_45_Gd_11_ indicate once more that these phases have to be much more stable than a mixture of their neighbouring phases.

### 3.3 Partial enthalpy of mixing of Cd in single-phase fields

**Fig. 4:**
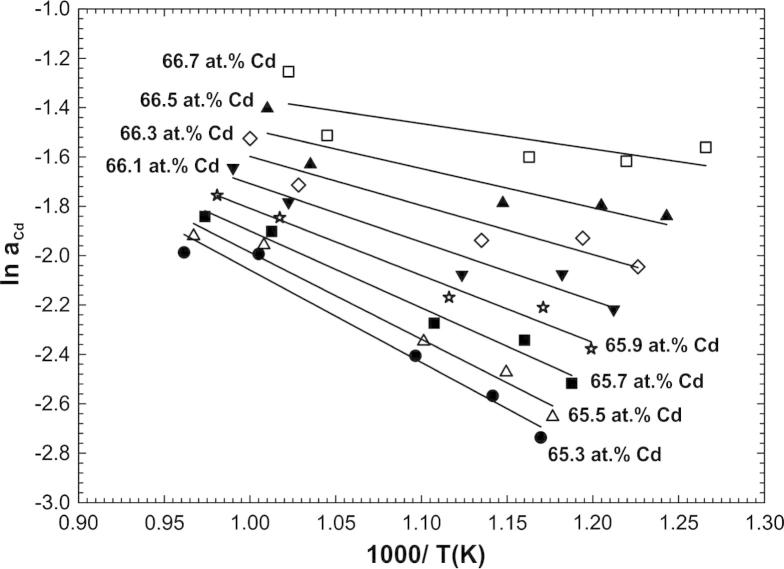
Natural logarithm of the Cd activity against reciprocal sample temperature for selected compositions in the homogeneity range of Cd_2_Gd.

Partial enthalpies of mixing of Cd were determined in the homogeneity ranges of Cd_2_Gd and Cd_45_Gd_11_ in the same manner as described above. For instance, for the evaluation of Cd_2_Gd, sample temperatures for selected compositions were obtained along the entire homogeneity range by interpolation from the equilibrium curves in Fig. 2. For these hypothetical samples, Cd activities were calculated according to Eqs. (1) and (3). Natural logarithms of these activities were plotted as a function of reciprocal temperature in Fig. 4. Applying the adapted Gibbs–Helmholtz equation (4), a linear regression was applied for data points of each selected composition in the entire homogeneity range of Cd_2_Gd. Although these data show some scatter it should be kept in mind that the data points are spaced in very narrow composition steps, and that small errors in composition and/or temperature in the equilibrium curves in Fig. 2 may lead to a noticeable shift in the ln *a*_Cd_ vs. 1/*T* data points.

**Fig. 5:**
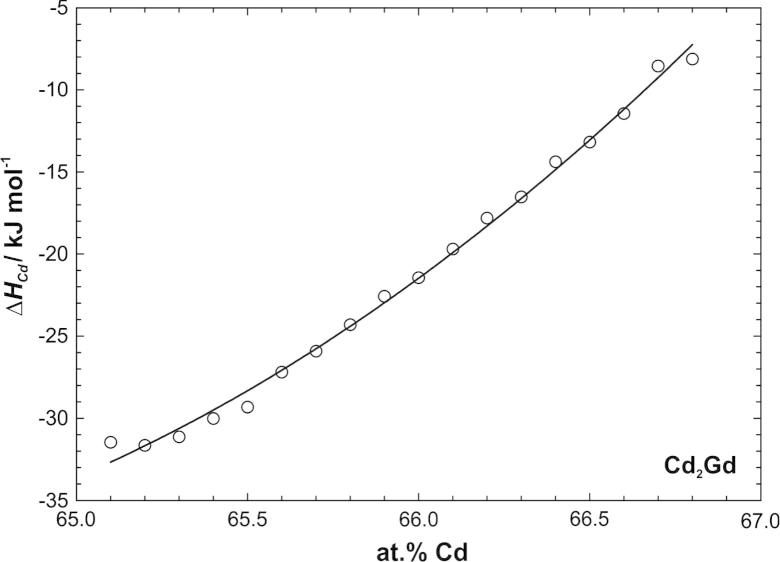
Partial molar enthalpy of Cd in the homogeneity range of Cd_2_Gd; standard state: Cd(l).

Values of $\Delta {\overset{¯}{H}}_{\text{Cd}}$ were directly calculated from the slopes of the straight lines in Fig. 4 and plotted against composition in Fig. 5. As can be seen, the partial enthalpy values vary over an extended range from −8 at the Cd-rich side to −32 kJ mol(Cd)^−1^ at the Gd-rich border of the homogeneity range, indicating once more the stability of this phase.

An analogous evaluation as given for Cd_2_Gd was carried out for the Cd_45_Gd_11_ phase. The corresponding values are included in Table 1. As discussed in detail in Ref. [18], the error in the partial enthalpies should be within ±5 kJ mol(at)^−1^ which was confirmed by an error analysis of the present data.

Partial enthalpy values within the homogeneity range of Cd_58_Gd_13_ could not be derived in the described way because of a limited number of samples, but were estimated from the neighbouring two-phase fields assuming a linear behaviour with composition between the values of the two-phase fields. The same procedure was employed for the Cd_3_Gd phase where no samples at all were available from the experiment (cf. Table 1).

### 3.4 Thermodynamic activity of Cd at 773 K

Thermodynamic activities of Cd for single- and two-phase fields were obtained at one particular temperature using an integrated form of the adapted Gibbs–Helmholtz equation:

(5) $$\ln a_{\text{Cd}}(773K)-\ln a_{\text{Cd}}(T_{\text{S}})=\frac{\Delta {\overset{¯}{H}}_{\text{Cd}}}{R}\cdot \left(\frac{1}{773}-\frac{1}{T_{\text{S}}}\right)$$

with the temperature in K and the partial enthalpy of Cd in J mol(Cd)^−1^. Accordingly, the activity values of all samples were converted to 773 K which represents a mean temperature of all sample temperatures *T*_S_. The required enthalpy values in the respective single- and two-phase fields were obtained according to Sections 3.2 and 3.3. As indicated in the previous chapter, the error in the partial enthalpies should be within ±5 kJ mol(at)^−1^ resulting in an error of at most ±0.15 in the value of ln *a*_Cd_. A detailed discussion concerning error sources and error limits in this isopiestic method was previously given by Ipser et al. [18].

**Fig. 6:**
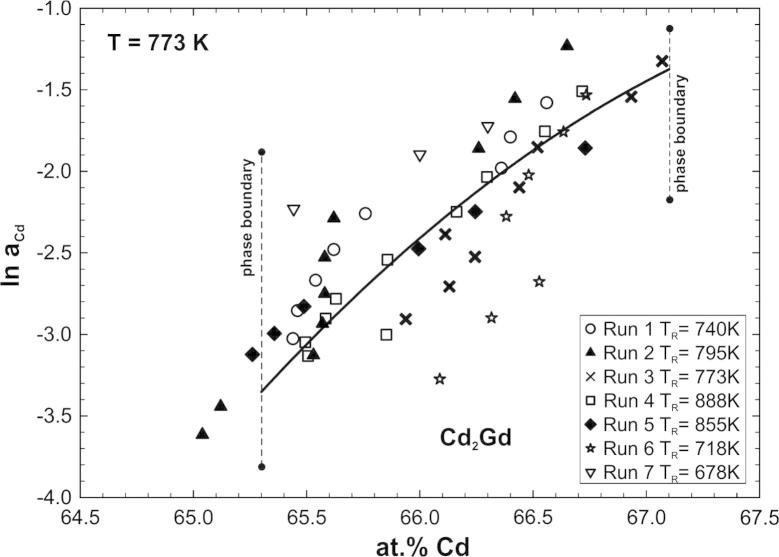
Natural logarithm of the Cd activity for Cd_2_Gd at 773 K; standard state: Cd(l). The symbols are the same as in Fig. 2.

As an example, ln *a*_Cd_ for Cd_2_Gd is shown in Fig. 6 as a function of composition at 773 K. The corresponding phase boundaries at this temperature were taken from Table 2. The data points were fitted with a polynomial function, leading to the best compatibility with the activity values in the adjacent two-phase fields.

As mentioned previously, numerous samples were located in the homogeneity range of Cd_45_Gd_11_, allowing a similar evaluation as it was done for Cd_2_Gd. The homogeneity range was estimated between 79.5 and 80.5 at.% Cd at 773 K and corresponding activity values of samples located within Cd_45_Gd_11_ were converted to 773 K. For the conversion of activity values to one particular temperature, enthalpy values of Cd as a function of composition were empirically fitted as described in 3.3. All enthalpy and activity values of Cd of single-phase samples of Cd_45_Gd_11_ are included in Table 1.

Thermodynamic activities of Cd at 773 K for the phases Cd_3_Gd and Cd_58_Gd_13_, given in Table 1, had to be converted from their corresponding sample temperatures with enthalpy values estimated by a linear interpolation between the values in the neighbouring two-phase fields, as discussed above.

The Cd activity at 773 K in the two-phase field Cd_6_Gd + L could not be obtained from the present measurements but was calculated from the results of Roshchina and Bayanov [14]. For this two-phase field a value of ln *a*_Cd_ = −0.04 was determined. The corresponding evaluation is described in detail in Section 3.5.

### 3.5 Integral Gibbs energy

As outlined above, Roshchina and Bayanov [14] determined thermodynamic activities of Gd in the liquid phase and the two-phase field Cd_6_Gd + L between 663 and 801 K. By means of a Gibbs–Duhem integration, the authors calculated the standard Gibbs energy of formation at the stoichiometric composition of Cd_6_Gd, given as −20.8 ± 0.6 kJ mol(at)^−1^, referred to Cd(s) and α-Gd(s) as standard states. The standard Gibbs energy of formation of Cd_6_Gd was re-calculated at 773 K to be about −16.1 kJ mol(at)^−1^, referred to Cd(l) and α-Gd(s). From the emf data at 775.15 K [14] a value for the thermodynamic activity of Gd could be obtained for the two-phase field Cd_6_Gd + L, namely ln *a*_Gd_ = −17.27. The corresponding solubility of Gd in liquid Cd obtained from the kink of the emf curve is given as 3.3 at.%. Assuming that the phase boundary Cd_6_Gd/Cd_6_Gd + L corresponds to the stoichiometric composition of Cd_6_Gd, i.e. there is no solid solubility of Cd within Cd_6_Gd at 773 K, an activity value of ln *a*_Cd_ = −0.04 was obtained for the two-phase field Cd_6_Gd + L .

**Fig. 7:**
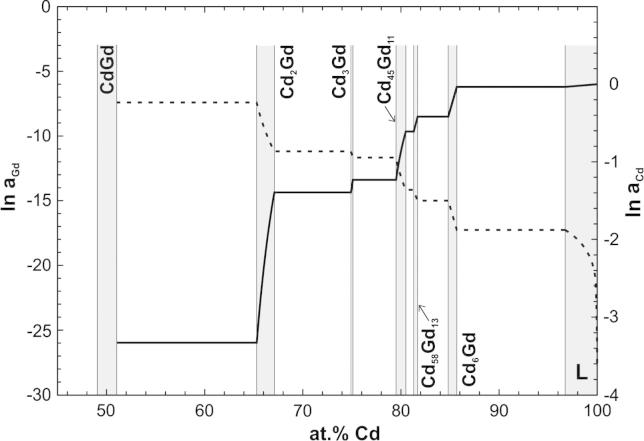
Natural logarithm of Cd and Gd activities against composition at 773 K, referred to Cd(l) and α-Gd(s) as standard states.

The activity value ln *a*_Gd_ = −17.27 served as an integration constant for a Gibbs–Duhem integration in the present study. The corresponding activity values of Cd and Gd in the composition range 51–100 at.% Cd are plotted as natural logarithms in Fig. 7. Once more it is indicated that the intermetallic compounds Cd_2_Gd and Cd_45_Gd_11_ have to be very stable, due to the marked variation of the activities across their homogeneity ranges.

**Fig. 8:**
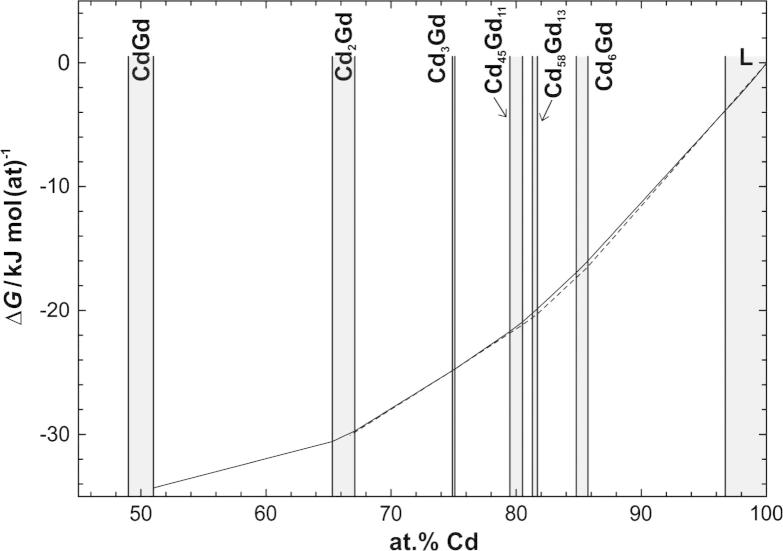
Integral Gibbs energy of formation against composition at 773 K, referred to Cd(l) and α-Gd(s) as standard states; dashed line refers to Kurata and Sakamura [17].

From the activity data for Cd and Gd, integral Gibbs energies were calculated for the respective composition range at 773 K and plotted in Fig. 8, referred to Cd(l) and α-Gd(s) as standard states. Gibbs energies of formation at the exact stoichiometric compositions of the phases Cd_58_Gd_13_, Cd_45_Gd_11_, Cd_3_Gd and Cd_2_Gd were obtained to be about −19.9, −21.1, −24.8, and −30.0 kJ g atom^−1^ at 773 K, respectively. Assuming an error of ±0.15 in the value of ln *a*_Cd_ at 773 K (as discussed above) and relying on the error limit given by Roshchina and Bayanov [14], the error in the integral Gibbs energy can be estimated to be in the range of ±1 kJ mol(at)^−1^ at about 85 at.% Cd, increasing to at most ±5 kJ mol(at)^−1^ around 50 at.% Cd.

The integral Gibbs energies of all intermetallic compounds were compared with those listed by Kurata and Sakamura [17] who made a CALPHAD-type optimization. The very good agreement in Fig. 8 proves that the assumptions that were used in the present Gibbs–Duhem integration are actually quite reasonable.

## 4 Summary

Seven isopiestic runs in total were carried out to measure vapour pressures of Cd in the system Cd–Gd between 693 and 1045 K. For this purpose, the intermetallic compound Cd_45_Gd_11_ was used as a master compound and equilibrated with Cd vapour at constant Cd vapour pressures between 2 and 137 mbar, respectively. Plotting sample temperatures against sample compositions, equilibrium curves were drawn (Fig. 2) and thermodynamic activity values of Cd were calculated for the composition range 51–86 at.% Cd. By employing an adapted Gibbs–Helmholtz equation partial molar enthalpies of mixing of Cd were obtained for the corresponding composition range, which were used to convert the activity values of Cd to a common average temperature of 773 K. With additional information from literature concerning a value for the activity of Gd in the two-phase field Cd_6_Gd + L [14], a Gibbs–Duhem integration was performed. The corresponding activity values of Cd and Gd are reported for the composition range 50–100 at.% Cd, see Fig. 7. From these data integral Gibbs energies were calculated and presented as a function of composition in Fig. 8. Gibbs energies of formation at the exact stoichiometric compositions of the phases Cd_58_Gd_13_, Cd_45_Gd_11_, Cd_3_Gd and Cd_2_Gd were obtained to be about −19.9, −21.1, −24.8, and −30.0 kJ g atom^−1^ at 773 K, respectively.

## Figures and Tables

**Table 1:** Experimental results of isopiestic experiments, standard state: Cd(l).

Sample no.	Cd (at.%)	*T*_S_ (K)	ln *a*_Cd_ (*T*_S_)	Phases	$\Delta {\overset{‾}{H}}_{\text{Cd}}/\text{kJ}\;\text{g}{\text{-atom}}^{-1}$	ln *a*_Cd_ (773 K)
Run 1: T_R_ = 740 K, 40 days						
1	84.11	763	−0.50	Cd_58_Gd_13_ + Cd_6_Gd	−15.5	−0.47
2	79.96	771	−0.67	Cd_45_Gd_11_	−15.5	−0.66
3	79.73	780	−0.85	Cd_45_Gd_11_	−21.1	−0.88
4	79.60	788	−1.01	Cd_45_Gd_11_	−25.7	−1.09
5	79.40	796	−1.17	Cd_3_Gd + Cd_45_Gd_11_	−26.2	−1.29
6	79.19	804	−1.32	Cd_3_Gd + Cd_45_Gd_11_	−26.2	−1.48
7	66.56	813	−1.49	Cd_2_Gd	−11.9	−1.58
8	66.40	822	−1.65	Cd_2_Gd	−14.7	−1.79
9	66.36	831	−1.81	Cd_2_Gd	−15.3	−1.98
10	65.76	839	−1.95	Cd_2_Gd	−24.9	−2.26
11	65.62	848	−2.11	Cd_2_Gd	−27.0	−2.48
12	65.54	856	−2.24	Cd_2_Gd	−28.2	−2.67
13	65.46	864	−2.38	Cd_2_Gd	−29.3	−2.85
14	65.44	872	−2.50	Cd_2_Gd	−29.6	−3.02
15	65.18	880	−2.63	Cd_2_Gd	−33.0	−3.09
16	63.57	888	−2.76	CdGd + Cd_2_Gd	−30.4	−3.37
17	48.94	921	−3.25	CdGd	–	–
18	48.67	940	−3.51	CdGd	–	–

Run 2: T_R_ = 795 K, 28 days						
1	85.00	800	−0.10	Cd_6_Gd	–	−0.34^a^
2	84.89	807	−0.23	Cd_6_Gd	–	−0.38^a^
3	80.50	817	−0.41	Cd_45_Gd_11_	−14.7	−0.97
4	79.86	828	−0.61	Cd_45_Gd_11_	−17.6	−1.23
5	79.69	841	−0.84	Cd_45_Gd_11_	−22.5	−1.56
6	66.65	855	−1.08	Cd_2_Gd	−10.3	−1.23
7	66.42	869	−1.31	Cd_2_Gd	−14.3	−1.55
8	66.26	883	−1.53	Cd_2_Gd	−17.1	−1.86
9	65.62	895	−1.71	Cd_2_Gd	−27.0	−2.29
10	65.60	907	−1.89	Cd_2_Gd	−27.3	−2.52
11	65.58	919	−2.07	Cd_2_Gd	−27.6	−2.75
12	65.57	929	−2.21	Cd_2_Gd	−27.7	−2.93
13	65.53	939	−2.35	Cd_2_Gd	−28.3	−3.13
14	65.12	948	−2.47	Cd_2_Gd	−33.8	−3.44
15	65.04	956	−2.58	Cd_2_Gd	−34.8	−3.62
16	63.98	964	−2.68	CdGd + Cd_2_Gd	−30.4	−3.62

Run 3: T_R_ = 773 K, 28 days						
1	82.28	793	−0.40	Cd_58_Gd_13_ + Cd_6_Gd	−15.5	−0.46
2	80.44	803	−0.59	Cd_45_Gd_11_	−13.9	−0.67
3	80.27	805	−0.64	Cd_45_Gd_11_	−12.9	−0.72
4	80.18	808	−0.69	Cd_45_Gd_11_	−13.1	−0.78
5	80.03	811	−0.73	Cd_45_Gd_11_	−14.5	−0.84
6	79.72	813	−0.78	Cd_45_Gd_11_	−21.6	−0.94
7	79.45	815	−0.81	Cd_45_Gd_11_	−32.3	−1.07
8	79.69	817	−0.86	Cd_45_Gd_11_	−22.6	−1.05
9	79.41	821	−0.92	Cd_45_Gd_11_	−34.1	−1.23
10	79.59	826	−1.01	Cd_45_Gd_11_	−26.3	−1.27
11	68.96	833	−1.13	Cd_2_Gd + Cd_3_Gd	−26.4	−1.43
12	67.07	842	−1.29	Cd_2_Gd	−2.5	−1.32
13	66.93	852	−1.47	Cd_2_Gd	−5.1	−1.54
14	66.52	863	−1.65	Cd_2_Gd	−12.6	−1.85
15	66.44	875	−1.84	Cd_2_Gd	−14.0	−2.10
16	66.11	885	−2.00	Cd_2_Gd	−19.5	−2.39
17	66.24	895	−2.16	Cd_2_Gd	−17.3	−2.52
18	66.13	903	−2.28	Cd_2_Gd	−19.2	−2.71
19	65.94	910	−2.38	Cd_2_Gd	−22.2	−2.91
20	61.97	917	−2.48	CdGd + Cd_2_Gd	−30.4	−3.23

Run 4: T_R_ = 888 K, 55 days						
1	66.72	975	−1.22	Cd_2_Gd	−9.1	−1.51
2	66.55	986	−1.35	Cd_2_Gd	−12.0	−1.76
3	66.30	995	−1.46	Cd_2_Gd	−16.4	−2.04
4	66.16	1004	−1.58	Cd_2_Gd	−18.7	−2.25
5	65.86	1013	−1.68	Cd_2_Gd	−23.5	−2.54
6	65.63	1021	−1.77	Cd_2_Gd	−26.9	−2.78
7	65.58	1027	−1.84	Cd_2_Gd	−27.5	−2.90
8	65.49	1034	−1.92	Cd_2_Gd	−28.8	−3.05
9	65.51	1040	−1.99	Cd_2_Gd	−28.6	−3.13
10	65.85	1045	−2.05	Cd_2_Gd	−23.6	−3.00

Run 5: T_R_ = 855 K, 55 days						
1	66.73	963	−1.59	Cd_2_Gd	−8.9	−1.86
2	66.24	971	−1.70	Cd_2_Gd	−17.3	−2.25
3	65.99	978	−1.78	Cd_2_Gd	−21.3	−2.48
4	65.49	984	−1.86	Cd_2_Gd	−28.9	−2.83
5	65.36	991	−1.94	Cd_2_Gd	−30.7	−2.99
6	65.26	996	−2.01	Cd_2_Gd	−32.0	−3.12
7	63.88	1001	−2.07	CdGd + Cd_2_Gd	−30.4	−3.14

Run 6: T_R_ = 718 K, 58 days						
1	85.04	735	−0.40	Cd_6_Gd	–	−0.32^a^
2	84.59	739	−0.50	Cd_58_Gd_13_ + Cd_6_Gd	−15.5	−0.38
3	84.54	743	−0.57	Cd_58_Gd_13_ + Cd_6_Gd	−15.5	−0.48
4	80.40	749	−0.72	Cd_45_Gd_11_	−13.5	−0.65
5	80.28	752	−0.79	Cd_45_Gd_11_	−12.9	−0.73
6	80.19	755	−0.84	Cd_45_Gd_11_	−13.0	−0.80
7	80.09	758	−0.90	Cd_45_Gd_11_	−13.7	−0.86
8	79.99	760	−0.96	Cd_45_Gd_11_	−15.0	−0.92
9	79.93	763	−1.01	Cd_45_Gd_11_	−16.0	−0.98
10	79.90	766	−1.07	Cd_45_Gd_11_	−16.6	−1.05
11	79.87	771	−1.18	Cd_45_Gd_11_	−17.3	−1.17
12	70.18	777	−1.30	Cd_2_Gd + Cd_3_Gd	−26.4	−1.32
13	66.73	787	−1.51	Cd_2_Gd	−8.8	−1.50
14	66.63	798	−1.71	Cd_2_Gd	−10.6	−1.71
15	66.48	809	−1.93	Cd_2_Gd	−13.3	−2.02
16	66.38	821	−2.14	Cd_2_Gd	−15.0	−2.28
17	66.53	842	−2.52	Cd_2_Gd	−12.5	−2.68
18	66.32	851	−2.67	Cd_2_Gd	−16.1	−2.90
19	59.77	859	−2.81	CdGd + Cd_2_Gd	−30.4	−3.29

Run 7: T_R_ = 678 K, 58 days						
1	84.74	693	−0.39	Cd_6_Gd	–	–b
2	84.67	695	−0.45	Cd_6_Gd	–	–b
3	84.58	699	−0.56	Cd_58_Gd_13_ + Cd_6_Gd	−15.5	−0.28
4	83.35	702	−0.61	Cd_58_Gd_13_ + Cd_6_Gd	−15.5	−0.36
5	81.43	707	−0.76	Cd_58_Gd_13_	−15.8^a^	−0.53
6	79.98	710	−0.81	Cd_45_Gd_11_	−15.2	−0.60
7	80.18	714	−0.92	Cd_45_Gd_11_	−13.1	−0.75
8	80.36	720	−1.05	Cd_45_Gd_11_	−13.2	−0.90
9	79.62	727	−1.23	Cd_45_Gd_11_	−25.0	−0.99
10	79.17	736	−1.43	Cd_3_Gd + Cd_45_Gd_11_	−26.2	−1.22
11	67.10	743	−1.60	Cd_2_Gd	−1.9	−1.78
12	66.30	752	−1.79	Cd_2_Gd	−16.4	−1.88
13	66.00	760	−1.96	Cd_2_Gd	−21.3	−2.17
14	65.44	773	−2.23	Cd_2_Gd	−29.5	−2.23

a Value was estimated by linear interpolation of the partial enthalpies/activity values of Cd in the adjacent two-phase fields.

b Samples are single-phase at *T*_S_ but two-phase (Cd_6_Gd + Cd_58_Gd_13_) at 773 K.

**Table 2:** Homogeneity ranges of the intermetallic compounds at 773 K estimated from the equilibrium curves; the phase boundary of the liquid was taken from Johnson [12].

Phase	Phase boundaries (at.% Cd)	Reference
CdGd	49.0–51.0	
Cd_2_Gd	65.3–67.1	
Cd_3_Gd	74.9–75.1	
Cd_45_Gd_11_	79.5–80.5	
Cd_58_Gd_13_	81.3–81.7	
Cd_6_Gd	84.8–85.7	
L	96.7–100	[12]

## References

[1] Olander D. (2009). Nuclear fuels – present and future. J. Nucl. Mater..

[2] Johnson I. (1988). The Thermodynamics of pyrochemical processes for liquid metal reactor fuel cycles. J. Nucl. Mater..

[3] Ackerman J.P. (1991). Chemical basis for pyrochemical reprocessing of nuclear–fuel. Ind. Eng. Chem. Res..

[4] Laidler J.J., Battles J.E., Miller W.E., Ackerman J.P., Carls E.L. (1997). Development of pyroprocessing technology. Progr. Nucl. Energy.

[5] Yamana H., Wakayama N., Souda N., Moriyama H. (2000). Systematics of the thermodynamic properties of trivalent f-elements in a pyrometallurgical bi-phase extraction system. J. Nucl. Mater.

[6] Moriyama H., Yamana H., Nishikawa S., Shibata S., Wakayama N., Miyashita Y., Moritani K., Mitsugashira T. (1998). Thermodynamics of reductive extraction of actinides and lanthanides from molten chloride salt into liquid metal. J. Alloys Comp..

[7] Kurata M., Sakamura Y., Hijikata T., Kinoshita K. (1995). Distribution behavior of uranium, neptunium, rare-earth elements (Y, La, Ce, Nd, Sm, Eu, Gd) and alkaline-earth metals (Sr, Ba) between molten LiCl–KCl eutectic salt and liquid cadmium or bismuth. J. Nucl. Mater..

[8] Reichmann T.L., Ipser H. (2014). Thermochemical investigations in the system cadmium–praseodymium relevant for pyrometallurgical fuel reprocessing metall. Mater. Trans. A.

[9] Skołyszewska-Kühberger B., Reichmann T.L., Ganesan R., Ipser H. (2014). Thermodynamic study of the cerium–cadmium system. CALPHAD.

[10] Reichmann T.L., Effenberger H.S., Ipser H. (2014). Experimental investigation of the Cd–Pr phase diagram. Plos One.

[11] Bruzzone G., Fornasini M.L., Merlo F. (1971). The gadolinium–cadmium system. J. Less-Common. Met..

[12] Johnson I. (1962). Rare Earth Research.

[13] Tang J., Gschneidner K.A. (1996). Physical metallurgy and magnetic behaviour of Cd-stabilized b.c.c. β-Gd alloys. J. Alloys Comp..

[14] Roshchina V.R., Bayanov A.P. (1981). Thermochemistry of gadolinium–cadmium alloy formation. J. Phys. Chem..

[15] Kurata M., Sakamura Y., Matsui T. (1996). Thermodynamic quantities of actinides and rare earth elements in liquid bismuth and cadmium. J. Alloys Comp..

[16] Y. Sakamura, T. Inoue, T.S. Storvick, L.F. Grantham, Characterizations of Rare Earths and Actinides in a Molten Salt/Liquid Cadmium System, in: 26th Symp. on Molten Salt Chem., Sapporo, 1995, p. 101.

[17] Kurata M., Sakamura Y. (2001). Thermodynamic assessment of systems of actinide or rare earth with Cd. J. Phase Equilib..

[18] Ipser H., Krachler R., Komarek K.L., Brodowsky H., Schaller H.-J. (1989). Thermochemistry of Alloys.

[19] Binnewies M., Milke E. (1999). Thermochemical Data of Elements and Compounds.

[20] Bruzzone G., Fornasini M.L., Merlo F. (1973). Rare earth intermediate phases with cadmium. J. Less-Common Met..

